# Control of Phasic Firing by a Background Leak Current in Avian Forebrain Auditory Neurons

**DOI:** 10.3389/fncel.2015.00471

**Published:** 2015-12-10

**Authors:** André A. Dagostin, Peter V. Lovell, Markus M. Hilscher, Claudio V. Mello, Ricardo M. Leão

**Affiliations:** ^1^Department of Physiology, School of Medicine of Ribeirão Preto, University of São PauloRibeirão Preto, Brazil; ^2^Department of Behavioral Neuroscience, Oregon Health and Science University, PortlandOR, USA; ^3^Brain Institute, Federal University of Rio Grande do NorteNatal, Brazil; ^4^Institute for Analysis and Scientific Computing, Vienna University of TechnologyVienna, Austria

**Keywords:** action potentials, leak current, potassium currents, zebra finch, birdsong

## Abstract

Central neurons express a variety of neuronal types and ion channels that promote firing heterogeneity among their distinct neuronal populations. Action potential (AP) phasic firing, produced by low-threshold voltage-activated potassium currents (VAKCs), is commonly observed in mammalian brainstem neurons involved in the processing of temporal properties of the acoustic information. The avian caudomedial nidopallium (NCM) is an auditory area analogous to portions of the mammalian auditory cortex that is involved in the perceptual discrimination and memorization of birdsong and shows complex responses to auditory stimuli We performed *in vitro* whole-cell patch-clamp recordings in brain slices from adult zebra finches (*Taeniopygia guttata*) and observed that half of NCM neurons fire APs phasically in response to membrane depolarizations, while the rest fire transiently or tonically. Phasic neurons fired APs faster and with more temporal precision than tonic and transient neurons. These neurons had similar membrane resting potentials, but phasic neurons had lower membrane input resistance and time constant. Surprisingly phasic neurons did not express low-threshold VAKCs, which curtailed firing in phasic mammalian brainstem neurons, having similar VAKCs to other NCM neurons. The phasic firing was determined not by VAKCs, but by the potassium background leak conductances, which was more prominently expressed in phasic neurons, a result corroborated by pharmacological, dynamic-clamp, and modeling experiments. These results reveal a new role for leak currents in generating firing diversity in central neurons.

## Introduction

The brain possesses an astonishing diversity of neuronal cells with firing properties that are specifically adapted for the roles they play in different aspects of computational processing. For example, the mammalian cerebral cortex contains distinct classes of GABAergic interneurons that exhibit firing patterns ranging from fast spiking to slow adapting, to intrinsic burst firing. Such characteristic electrophysiological features are thought to be adaptations for the computational demands required by different cortical microcircuits (Ascoli et al., 2008; Battaglia et al., 2013). In sensory systems, firing diversity allows for an enhanced representation of complex stimuli by expanding the range of responses to varied inputs (Padmanabhan and Urban, 2010). For example, firing heterogeneity in the cochlear nuclei allows individual neurons to extract information from complex auditory stimuli in both the frequency and time domains (Young and Oertel, 2004; Yang and Feng, 2007).

Not surprisingly, heterogeneity in firing properties can be correlated with specific combinations of ion channels whose regulated expression varies considerably across neuronal populations (Llinas, 1998). In particular, voltage-activated potassium channels, including both inactivating and delayed rectifier types, have received considerable attention for the roles they play in determining the neuronal excitability and firing patterns (Llinas, 1998; Dodson and Forsythe, 2004). In the auditory brainstem, phasic firing is controlled by the expression of Kv1 low-voltage-activated potassium currents (VAKCs; Manis and Marx, 1991; Brew and Forsythe, 1995; Dodson et al., 2002; Svirskis et al., 2002; Fukui and Ohmori, 2003), while Kv3 high-threshold activated potassium currents are important for shortening the AP and high-frequency firing (Fukui and Ohmori, 2003; Macica et al., 2003). In contrast, subthreshold conductances, like potassium inwardly rectifiers (*I*_Kir_) and hyperpolarization activated cation channels (*I*_h_), and background (“leak”) conductances are generally thought to be mainly associated with the regulation of passive membrane properties and resting membrane potential (RMP; Day et al., 2005; Biel et al., 2009; Enyedi and Czirják, 2010; Hibino et al., 2010; Leao et al., 2012), and thus have received less attention with respect to cellular firing properties.

The caudomedial nidopallium (NCM) occupies a position in the auditory forebrain circuitry of birds that is analogous to supragranular layers of the auditory cortex of mammals (Vates et al., 1996; Mello et al., 1998). Based on extensive molecular and electrophysiological studies in zebra finches (*Taeniopygia guttata*), the NCM has been shown to be involved in the perceptual processing, discrimination, and memorization of birdsong (Mello et al., 1992; Pinaud et al., 2004; Bolhuis and Gahr, 2006; Phan et al., 2006; Gobes and Bolhuis, 2007; London and Clayton, 2008; Woolley and Doupe, 2008; Moorman et al., 2011). Despite its relevance for songbird processing and a recent analysis of the potassium channels genes expressed in the zebra finch brain (Lovell et al., 2013), no information is available regarding the electrophysiological properties of these neurons. In order to fill this gap, we performed an electrophysiological recordings of NCM neurons and found that half of NCM neurons fire phasically (only one action potential, AP) when depolarized, while the rest fire trains of two or more APs. They have similar RMP and VAKCs but phasic neurons have a smaller membrane input resistance. Surprisingly we found that the phasic firing is governed by a relatively large background leak conductance that directly influences intrinsic excitability, constraining firing to a single AP. To our knowledge, this is the first report of a direct role for a leak-type current in the generation of neuronal firing diversity.

## Materials and Methods

### Brain Slices and Whole-cell Patch-clamp

All procedures were conducted in accordance with the Ethics Committee on Animal Experimentation of the School of Medicine of Ribeirão Preto – University of São Paulo and the Institutional Animal Care and Use Committee at OHSU. For electrophysiological experiments a total of 87 adult male and female zebra finches (*T. guttata*) were used. Brain slices were prepared as described previously (Dagostin et al., 2012). Briefly, birds were sacrificed by decapitation, their skulls opened and their brains removed into ice-cold cutting solution containing in mM: 87 NaCl, 2.5 KCl, 25 NaHCO_3_, 1.25 NaH_2_PO_4_, 75 sucrose, 25 glucose, 3 myo-Inositol, 0.2 CaCl_2_, 7 MgCl_2_, 0.4 ascorbic acid, 2 sodium pyruvate; ∼345mOsm/kg H_2_O, pH 7.4 when saturated with carbogenic mix (95% O_2_, 5% CO_2_). Parasagittal slices (250 μm thick) were cut starting from the midline using a vibratome (Vibratome 1000 Plus, Vibratome, St Louis, MO, USA). Although the NCM does not have a visually identifiable lateral boundary, previous studies based on song-inducible gene expression (Mello and Clayton, 1994) indicated that the lateral-most boundary of NCM is located ∼1 mm from the midline. We therefore restricted our recordings to cells present in the first three slices (∼750 μm). Slices were incubated at least for 30 min in cutting solution at room temperature prior to recording.

For electrophysiological recordings, slices were transferred to a recording chamber mounted on the stage of an upright microscope (Olympus BX51WI) and continuously perfused with an avian artificial cerebral spinal fluid (aCSF; Bottjer, 2005) containing in mM: 135 NaCl, 2.5 KCl, 25 NaHCO_3_, 1.25 NaH_2_PO_4_, 25 glucose, 3 myo-inositol, 2 CaCl_2_, 1 MgCl_2_, 1 ascorbic acid, 2 sodium pyruvate; ∼ 310 mOsm/kg H_2_O, pH 7.4 when saturated with carbogenic mix(95% O_2_, 5% CO_2_). Single neurons were visualized with infrared-differential interference contrast (IR-DIC) optics.

All recordings were made at room temperature (∼25°C) with a HEKA EPC10 amplifier (HEKA Electronics, Germany). Some recordings were made at 32–35°C with an inline heather (AutoMate Scientific, Berkely, CA, USA) but we observed that NCM neurons in slices did not endure much time in temperatures above 30°C. Pipettes were pulled from borosilicate glass (BF-150-86-10, Sutter Instruments, Novato, CA, USA) with a horizontal puller (P-97. Sutter Instruments), and had a measured tip resistance of 3–6 MΩ when backfilled with an internal solution containing potassium gluconate as the main source of K^+^ (in mM: 130 K-gluconate, 5 EGTA, 10 HEPES, 20 KCl, 2 ATP-Mg, 0.2 GTP-Na, 5 phosphocreatine-Na; ∼320 mOsm, pH 7.3 corrected with KOH). After whole-cell recording configuration was attained, series resistance and capacitance were electronically canceled, and cells that had a series resistance >30 MΩ were removed from further analysis (mean series resistance 20.25 ± 0.9 MΩ, *N* = 56. Not significantly different among the three neuronal types. *P* = 0.78, One-way ANOVA). Data collection was initiated after at least 5 min under whole-cell configuration and care was taken to observe any spontaneous changes in neuronal firing during recordings. All neurons that showed changes in either spontaneous firing and/or input resistance during the recording period were removed from the analysis. Neurons with signs of poor seal or health, with RMP above –40 mV and/or APs with a peak smaller than 0 mV, were not considered for analysis.

In our initial experiments we used a mammalian aCSF and observed that some neurons changed their firing behavior from tonic/transient to phasic during the first 5–10 min of recording. This effect was accompanied by a marked decrease in input resistance. However, by switching to an avian-aCSF with higher osmolality than the mammalian aCSF (by adding 10 mM NaCl; Bottjer, 2005) we found that this effect was mostly eliminated.

All signals were low-pass filtered at 3 kHz (Bessel), and acquired at 10 kHz in voltage-clamp mode and 50 kHz in current-clamp mode. For current clamp recordings neurons were kept at their RMP, however, for experiments involving the application of Ba^2+^ 5 mM, we applied a small negative DC current to restore neurons to their normal RMP. Membrane input resistance (*R*_i_) was measured by applying 2–3 negative current injection pulses and measuring the steady-state membrane potential. Because the *R*_i_ varied considerably among neurons, we varied both the absolute range and relative step size accordingly. APs were evoked by square-wave depolarizing current injections (500–3000 ms) at several current levels. The depolarization sag of the membrane was measured during hyperpolarizations to –100 to –120 mV.

Voltage-activated and leak potassium currents were recorded in the presence of tetrodotoxin (TTX; 1 μM) to block voltage-activated sodium current (unless we were measuring AP firing concomitantly). Voltage-activated potassium currents were elicited by 1 s steps of 20 mV, from a holding potential of –70 mV. A P/-4 leak-subtracting protocol was used to subtract leak and capacitive currents from voltage-dependent currents. Sub-threshold membrane currents were recorded in voltage-clamp mode starting from a holding potential of –50 mV by applying hyperpolarizing voltage pulses of 1 s (5 s interval) ranging from –50 mV to –110/–120 mV in steps of –20 or –10 mV. Sequential application of BaCl_2_ (0.2 mM) and CsCl (3 mM) was used to inhibit the *I*_Kir_ and *I*_h_, respectively. Potassium leak currents were inhibited by perfusion of BaCl_2_ 5 mM. The BaCl_2_ 5 mM sensitive current was identified as the potassium leak current.

All solutions were prepared with ultrapure water (resistivity > 18 M ohm) and analytical grade salts. TTX was obtained from Tocris Bioscience (Bristol, UK), and all other drugs were obtained from Sigma (St. Louis, MO, USA).

#### Dynamic-clamp

We simulated a background potassium leak current using the Real Time Application Interface for Linux-based (RTAI^1^) dynamic clamp (Hilscher et al., 2013). Two computers were used, one for data acquisition running PatchMaster (Heka Elektronik), and a second ‘dynamic-clamp’ computer that reads voltage from the patch-clamp amplifier and generates current commands in real-time every 100 μs. The ‘dynamic-clamp’ computer is an x86 architecture computer (Pentium 4) with a PCI-6036E data acquisition card (National Instruments) for reading voltage and generating current commands to the clamped neuron. The real-time dynamic clamp software was written (by Dr. R.N. Leão, Federal University of Rio Grande do Norte, Brazil) in GNU-C, and routines for data acquisition were programmed using the Linux Control and Measurement Device Interface (COMEDI^2^). Values for Ba^2+^-sensitive leak currents were obtained for each neuron by applying two steps of –10 mV from a holding potential of –70 mV; these currents were modeled as ohmic conductances with reversal potentials calculated by linear extrapolation.

### Neuronal Simulations

A single compartment model (similar to Rothman and Manis, 2003) was implemented where changes in membrane potential are governed by sodium (*I*_Na_), potassium (*I*_K_) and leak currents (*I*_L_), and injected current (*I*_app_ = 120 pA), as described by the equation:

$$C*dV/dt=-I_{Na}-I_{K}-I_{L}+I_{app}$$

where *C*, *V*, *E*, *g*, and *I* denote the capacitance density (1 μF/cm^2^), voltage (mV), reversal potential (mV; *E*_Na_ = 50, *E*_K_ = –94, *E*_L_ = –77), conductance density (mS/cm^2^; *g*_Na_ = 91.66, *g*_K_ = 20.83, *g*_L_ = 0.1667) and current density (μA/cm^2^), respectively. The kinetics of the transient sodium current were modeled based on a Hodgkin–Huxley type sodium current (Hodgkin and Huxley, 1952), as given by:

$$I_{Na}=g_{Na}*m^{3}*h*\left(V-E_{Na}\right)$$

The potassium current (*I*_K_) was modeled as a non-inactivating delayed rectifier (similar to Kanold and Manis, 2001) with the experimentally obtained values for *V*_1/2_ = 8.4 mV, *k* = 18.5 and τ_a_ = 3.2 ms using the Hodgkin–Huxley formalism:

$$I_{K}=g_{K}dr*a^{2}*\left(V-E_{K}\right)$$

Its voltage-dependence of the activation was defined by the Boltzman equation:

$$a\infty =1/\left(1+\exp\left((V_{1/2}-V)/K\right)\right)$$

And its time-dependence of activation by the first-order differential equation below:

$$da/dt=(a\infty -a)/\tau_{a}$$

To investigate how different K-current types might affect the ability of our simulated neuron to fire phasically or repetitively, we added to the non-inactivating delayed rectifier (*I*_K_) either a low-threshold K-current (*I*_Klt_, *g*_Klt_ = 16.67; similar to Rothman and Manis, 2003), a high-threshold K-current (*I*_Kht_, *g*_Kht_ = 12.50; similar to Rothman and Manis, 2003), or a fast-inactivating K-current (*I*_Ka_, *g*_Ka_ = 10.42; similar to Kanold and Manis, 2001) consisting of the following equations:

$$I_{Klt}=g_{Klt}*w^{4}*z*\left(V-V_{K}\right)$$

$$w_{\infty }={\left(1+\exp\left(-(V+48)/6\right)\right)}^{-1/4}$$

$$z_{\infty }=(1-\zeta )*{\left(1+\exp\left((V+71)/10\right)\right)}^{-1}+\zeta \,with\zeta =0.5$$

$$\tau_{w}=100*(6*\exp\left((V+60)/6\right))+16*{\exp\left(-\left((V+60)/45\right)\right)}^{-1}+1.5$$

$$\tau_{z}=1000*{\left(\exp((V+60)/20)+\exp\left(-(V+60)/8\right)\right)}^{-1}+50$$

$$I_{Kht}=g_{Kht}*(\varphi *n^{2}+(1-\varphi )*p)*(V-V_{K})\;with\,\varphi =0.85$$

$$n_{\infty }={\left(1+\exp\left(-(V+15)/5\right)\right)}^{-1/2}$$

$$P_{\infty }={\left(1+\exp\left(-(V+23)/6\right)\right)}^{-1}$$

$$\tau_{n}=100*{\left(11*\exp((V+60)/24)+21*\exp\left(-(V+60)/23\right)\right)}^{-1}+0.7$$

$$\tau_{p}=100*{\left(4*\exp((V+60)/32)+5*\exp\left(-(V+60)/22\right)\right)}^{-1}+5$$

$$I_{Ka}=g_{Ka}*m_{F}^{4}*h_{F}*\left(V-V_{K}\right)$$

$$m_{F\infty }={\left(1+\exp\left(-(V+53)/25.8\right)\right)}^{-1}$$

$$h_{F\infty }={\left(1+\exp\left((V+89.6)/6.7\right)\right)}^{-1}$$

$$\tau_{mF}={\left(0.15*\exp((V+57)/10)+0.3*\exp\left(-(V+57)/10\right)\right)}^{-1}+0.5$$

$$\tau_{hF}={\left(0.015*\exp((V+87)/20)+0.03*\exp\left(-(V+87)/20\right)\right)}^{-1}+10$$

The leak current (*I*_L_) had a conductance between 2 and 7.4 nS (or 0.1667 and 0.6167 mS/cm^2^), thus the passive time constant given by τ_0_ = C/gL, varied between ∼6 ms and ∼1.5 ms. *I*_L_ was modeled according to the equation:

$$IL=g_{L}\left(V-E_{L}\right)$$

All simulations were performed using the ordinary differential equation solver in MATLAB R2013a (Mathworks).

### Data Analysis

Analysis was performed in Igor Pro (Wavemetrics, Lake Oswego, OR, USA) using custom routines and Patcher’s Power Tools macros (PPT; Max-Planck Institute for Biophysical Chemistry, Gottingen, Germany). Voltage–Current (VI) and Current–Voltage (IV) relationships were constructed based on the final 100 ms of the waveforms. *R*_i_ was calculated from the slope of the VI curves and membrane conductances from the slopes of the IV curves, both in the potentials negative to –60 mV. The membrane time constant (τ_m_) was calculated by fitting a single exponential function to the decay of the repolarization phase of the membrane potential. The activation time constant (τ_a_) of voltage-activated potassium currents was measured by fitting a single exponential to the activation phase of the current. The AP threshold was defined as the point where the first derivative (*dV/dt*) of the AP reached the value of two times the standard deviation of the baseline. First-spike latency was calculated as the time between the start of the stimulus and the AP threshold, elicited at the minimum current value required to elicit an AP. The half-width (HW) of the AP in msec was the duration at the point of half the amplitude measured from the threshold to the peak. Rheobase was defined as the minimum amount of current needed to reach AP threshold from the RMP when applying current steps. All membrane potential measurements were corrected by a measured liquid junction potential (Neher, 1992) of 10 mV. Data is presented as mean ± SEM. All statistical analyses and graphs were made using Prism 5.0 (GraphPad, LaJolla, CA, USA) and Igor Pro (Wavemetrics, Lake Oswego, OR, USA).

## Results

**FIGURE 1:**
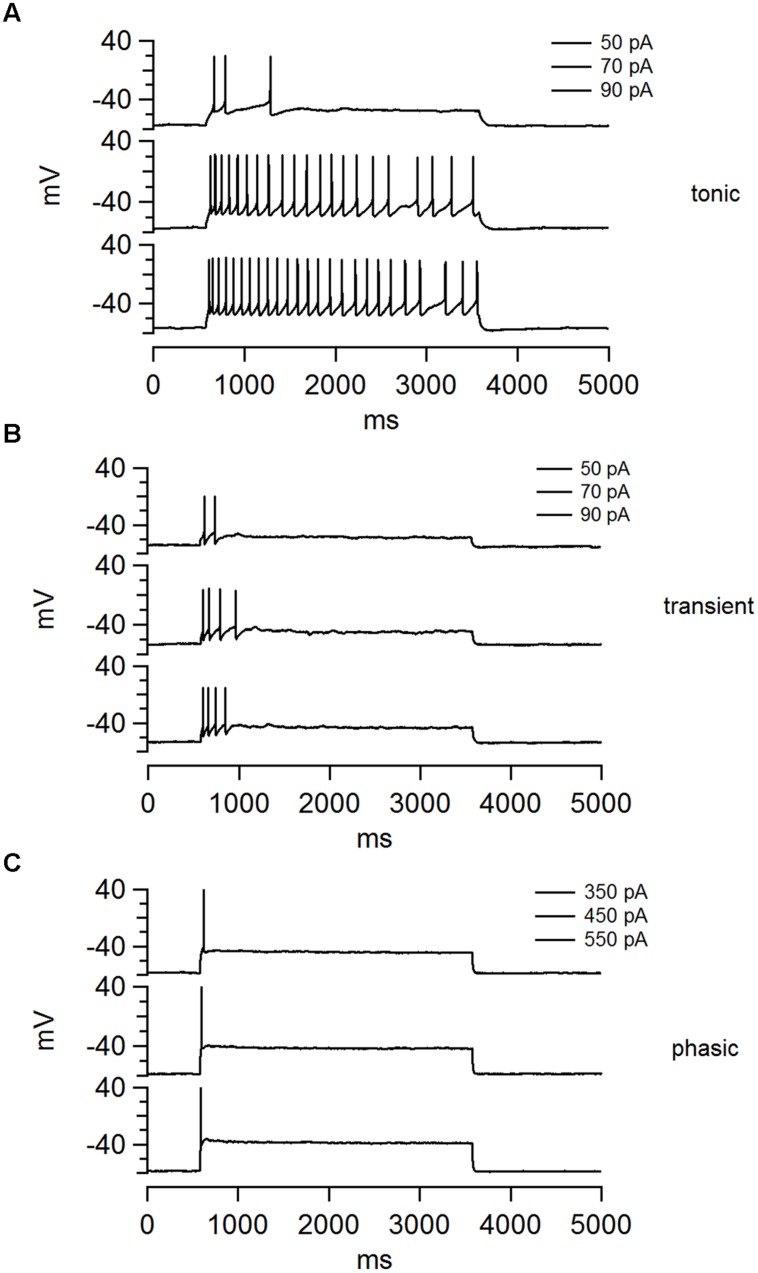
**The NCM contains three distinct classes of neurons that can be separate on the basis of their firing patterns. (A–C)** Examples of voltage traces for each the three cell types in NCM elicited by three current steps: **(A)** tonic, **(B)** transient, and **(C)** phasic. The current magnitude applied in each trace is shown at the top.

Electrophysiological recordings in current clamp mode, from randomly selected NCM neurons (162 neurons from 87 birds), revealed three distinct classes of neurons that could be easily classified based on their patterns of AP firing elicited by square pulses of depolarizing current (**Figure 1**). We refer to these cell types as having tonic, transient, and phasic firing patterns (representative traces are shown in **Figure 1**). Tonic firing neurons (**Figure 1A**) are defined by their ability to fire a continuous train of APs throughout the period of stimulation. These neurons typically respond to small injections of depolarizing current with a single AP, or a short transient train of APs, but become tonic with increasing current magnitude (**Figure 1A**). In contrast, transient firing neurons (**Figure 1B**) fire a short train of two or more APs, but stop firing before the termination of the stimulus. The number of APs can increase as a function of the magnitude of current injection, but the response never becomes completely tonic (**Figure 1B**). Finally, phasic firing neurons (**Figure 1C**) fire just a single AP in response to sustained depolarization, independent of the amount of injected current. The most frequently recorded neurons were phasic (*n* = 75; 49%), followed by transient (*n* = 49; 32%), and tonic firing types (*n* = 30; 19%). We note that only three of the 162 recorded neurons for this study exhibited spontaneous firing at RMP, and all three were tonic.

Both tonic and transient neurons showed spike accommodation during the stimulation period. Typically the inter-spike interval became longer, eventually reaching a plateau. On average these neurons fired initially at 22.1 ± 2.5 Hz, and then stabilized to 9.7 ± 0.7 Hz by the sixth AP (*n* = 22). We also observed that individual tonic and transient neurons produced APs with variable waveforms, after hyperpolarizations and spike frequencies, showing that these are a heterogeneous group of neurons. However, we did not attempt to further classify them based on these differences. In stark contrast, most phasic neurons showed very stereotyped firing behavior that was marked by APs with very similar waveforms.

**FIGURE 2:**
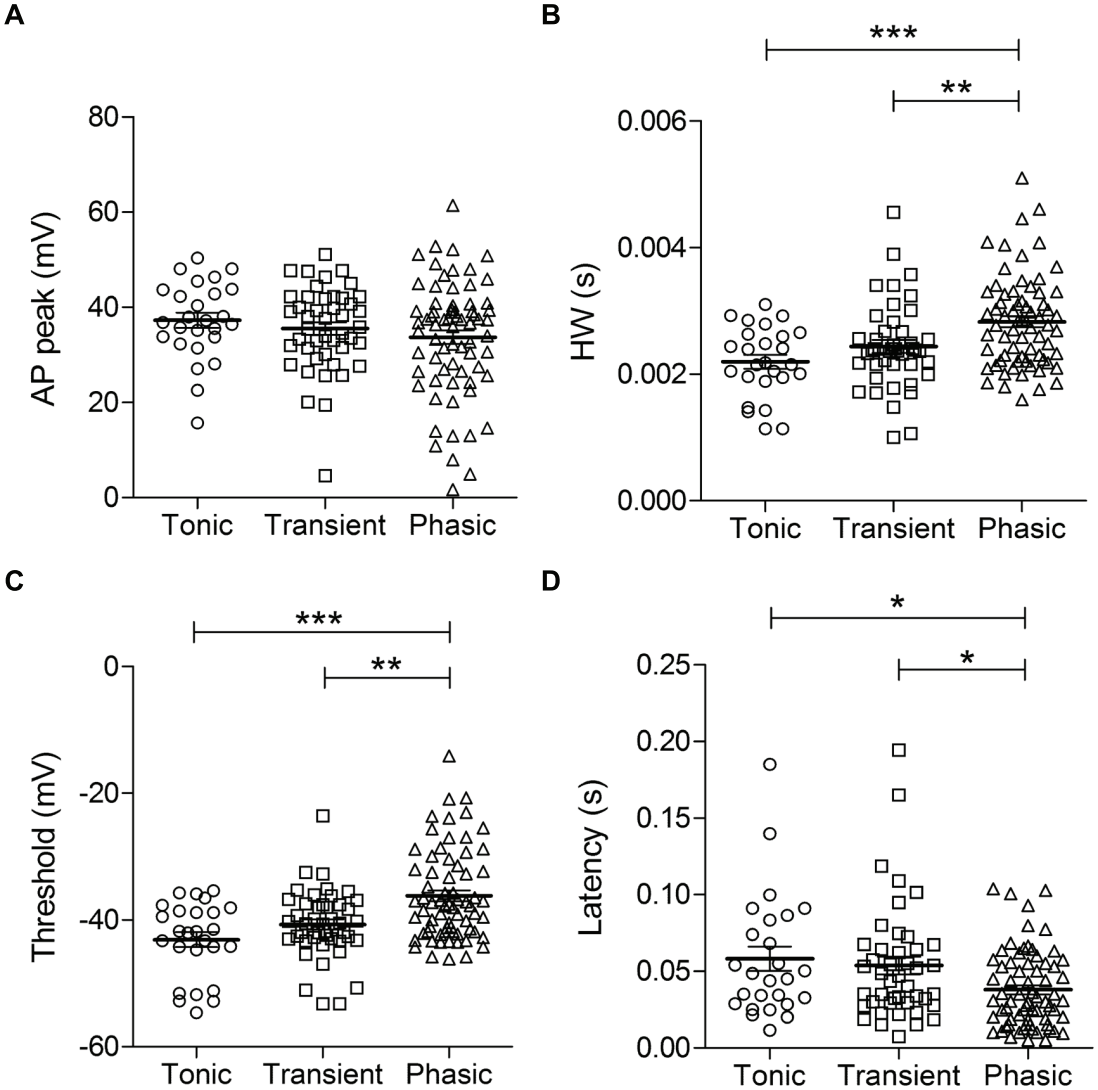
**Action potential (AP) parameters in the three neuronal types.** AP peak **(A)**, half-width **(B)**, threshold **(C)**, and first-spike latency **(D)** of tonic, transient, and phasic neurons. Scatters and mean ± SEM (^∗^*p* < 0.05; ^∗∗^*p* < 0.005; ^∗∗∗^*p* < 0.001). *n* = 26, 45, and 70, respectively.

Action potential waveforms did not significantly differ with regards to peak amplitudes (tonic: 37.3 ± 1.6 mV, *n* = 26; transient: 35.5 ± 1.3 mV, *n* = 45; phasic: 33.7 ± 1.4 mV, *n* = 70; *p* = 0.29, One-way ANOVA; **Figure 2A**). But phasic neurons had a broader HW compared to the other cell types (tonic: 2.2 ± 0.1 ms, *n* = 26; transient: 2.4 ± 0.1 ms, *n* = 44; phasic: 2.8 ± 0.1 ms, *n* = 69; *p* = 0.0001, One-way ANOVA, Newman–Keuls multiple comparisons test; **Figure 2B**). Furthermore, the mean AP threshold was significantly higher in phasic neurons than in the other cell types (tonic: –43.1 ± 1.2 mV, *n* = 26; transient: –40.7 ± 0.8 mV, *n* = 45; phasic: –36.2 ± 0.9 mV, *n* = 69; *p* < 0.0001, One-way ANOVA, Newman–Keuls multiple comparisons test; **Figure 2C**), and these neurons had a significantly shorter first-spike latency (tonic: 58.2 ± 7.9 ms, *n* = 26; transient: 53.8 ± 5.7 ms, *n* = 44; phasic: 38.0 ± 3.0 ms, *n* = 69; *p* = 0.012, One-way ANOVA, Newman–Keuls multiple comparisons test; **Figure 2D**).

### Membrane Input Resistance differs Significantly across NCM Cell Types

**FIGURE 3:**
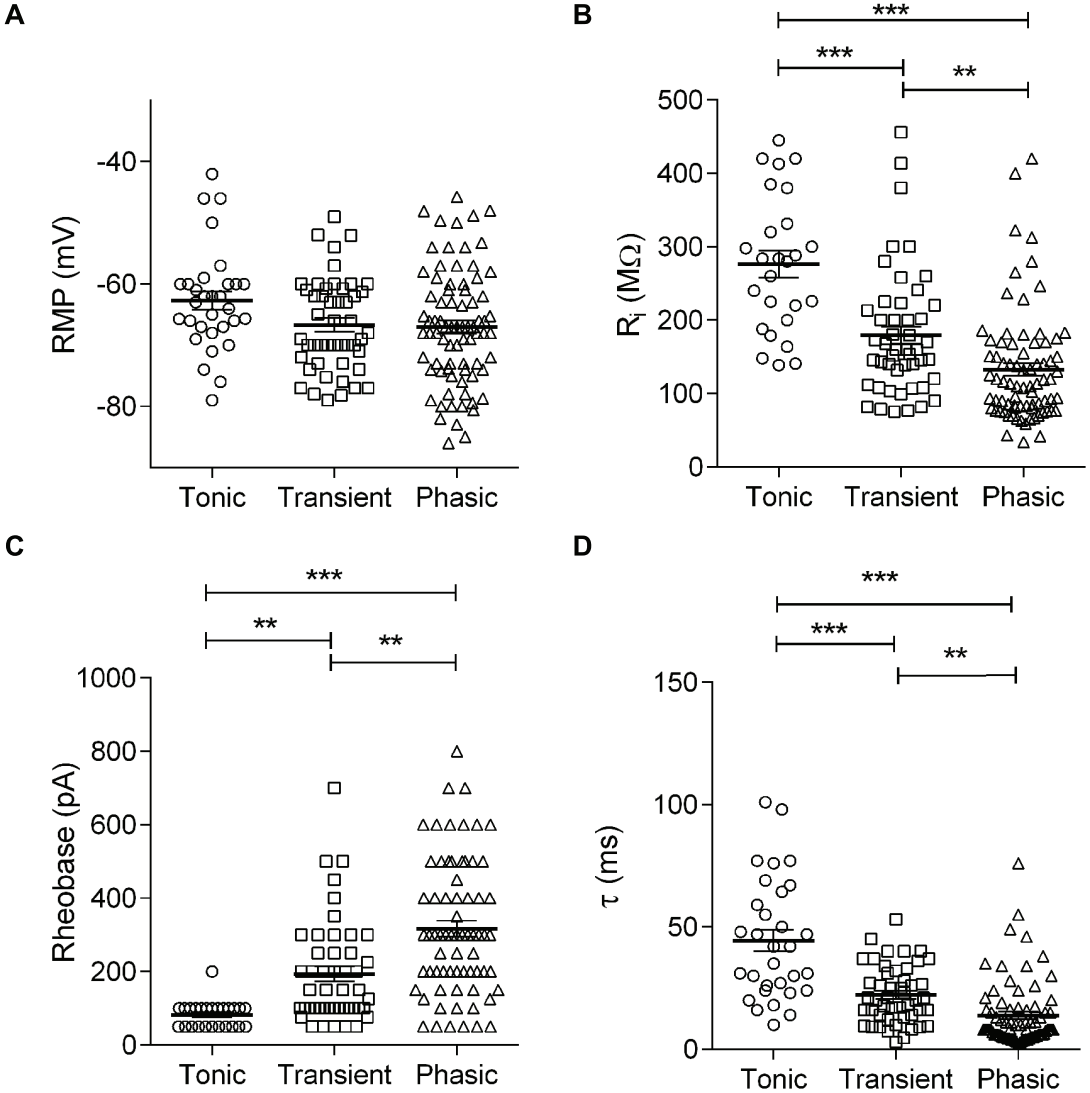
**Membrane passive properties in the three neuronal types: Membrane resting potential **(A)**, input resistance **(B)**, rheobase **(C)**, and time constant **(D)** of tonic, transient, and phasic neurons.** Scatters and mean ± SEM (^∗∗^*p* < 0.005; ^∗∗∗^*p* < 0.001). *n* = 26, 48, and 73, respectively, in **(A–C)** and 25, 47, and 62, respectively in **(D)**.

In the auditory system, the combination of elevated AP threshold, short first-spike latency and improved firing precision observed in the phasic neurons is characteristic of neurons with low membrane input resistance (*R*_i_; Oertel, 1999). We therefore compared the membrane *R*_i_, and other passive membrane properties for the three basic neuronal types in NCM. We did not detect significant differences in mean RMP (tonic: –62.7 ± 1.5 mV, *n* = 31; transient: –66.7 ± 1.1 mV, *n* = 48; phasic: –66.8 ± 1.1 mV, *n* = 73; *p* = 0.07, One way ANOVA; **Figure 3A**), ruling out the possibility that phasic or tonic firing could be related to different RMP levels. On the other hand, we did find significant differences in membrane *R*_i_. Specifically, tonic firing neurons had a significantly higher *R*_i_ than transient and phasic neurons (tonic: 276.1 ± 18.3 MΩ, *n* = 26; transient: 179.2 ± 12.4 MΩ, *n* = 48; phasic: 125.0 ± 8.2 MΩ, *n* = 73; *p* < 0.0001, One way ANOVA, Newman–Keuls multiple comparisons test; **Figure 3B**). Since the smaller input resistance would be expected to affect not only the membrane response to current, but also the rate of response to hyperpolarizing inputs, we also measured the membrane time constant (τ) by fitting a single exponential function to the decay phase of the membrane potential in response to hyperpolarizing pulses. As predicted, tonic neurons were found to have a significantly longer average τ value (44.5 ± 4.4 ms; *n* = 31) than transient (22.3 ± 1.7 ms, *n* = 42) and phasic neurons (13.9 ± 1.6 ms, *n* = 70; *p* < 0.0001, One-way ANOVA, Newman–Keuls multiple comparisons test; **Figure 3D**).

A smaller input resistance can be attributed to neurons with large cell bodies and/or a better preserved dendritic arbor after slicing. We therefore measured the membrane capacitance (*C*_m_) of each cell type, which is proportional to the cell membrane’s area, using the integral of the capacitive transients evoked by a 10 mV depolarization. We did not find significant differences in membrane capacitance between cell types (tonic: 70.2 ± 20 pF, *n* = 6; transient: 51.8 ± 10 pF, *n* = 13; phasic: 41.9 ± 3.8 pF, *n* = 27; *p* = 0.11, One-way ANOVA), suggesting that the smaller input resistance of phasic neurons is not a consequence of larger soma or dendritic arbors in these neurons.

Because of the temperature sensitivity of NCM neurons we performed the experiments at room temperature (25°C). But since some channels that can affect input resistance, like the TREK-1, TREK-2, and TRAAK potassium leak channels, have a large sensitivity to increasing temperatures above 24°C (Maingret et al., 2000; Kang et al., 2005), we compared the firing modes and membrane input resistance of neurons recorded at room temperature with neurons recorded at 35°C. We managed to obtain stable recordings from 26 neurons (10 phasic, 10 transient, and 6 tonic) at 35°C and we found not only the same basic types, but the same gradient of membrane input resistances (tonic > transient > phasic). However, while the membrane input resistance of phasic and transient neurons were similar at room temperature and at 35°C (Phasic: 125 ± 8 vs. 151 ± 12 MΩ, room temperature and 35°C, respectively, *P* = 0.25, *t*-test; Transient 179 ± 12 vs. 212 ± 22 MΩ, room temperature and 35°C, respectively, *P* = 0.25, *t*-test), tonic neurons had significantly bigger input resistances at 35°C (276 ± 18 vs. 486 ± 53 MΩ, room temperature and 35°C, respectively, *P* = < 0.0001, *t*-test). So we conclude that the differences in membrane input resistance among the three neuronal types seen at room temperature reflect the physiological conditions, but tonic neurons present an even bigger membrane input resistance at more physiological temperatures.

The observed differences in *R*_i_ not only affect the speed of the membrane response, but also potentially affect the excitability of NCM neurons. Therefore, we next analyzed rheobase, a parameter that reflects neuronal excitability. Neurons with low *R*_i_ values typically need large synaptic currents to reach AP threshold and vice versa. Based on *R*_i_ measurements, we predicted that phasic neurons would need significantly more current to reach the threshold for firing an AP than tonic neurons. As predicted, we found that tonic neurons had, on average, the lowest rheobase values (82.0 ± 7.0 pA, *n* = 25), followed by transient (193.1 ± 20.5 pA, *n* = 47) and phasic neurons (316.7 ± 22.1 pA, *n* = 69), the latter having a mean rheobase nearly four times greater than in tonic neurons (*p* < 0.0001; One-way ANOVA, Newman–Keuls multiple comparisons test, **Figure 3C**).

### Subthreshold Currents are Larger in Phasic Neurons

**FIGURE 4:**
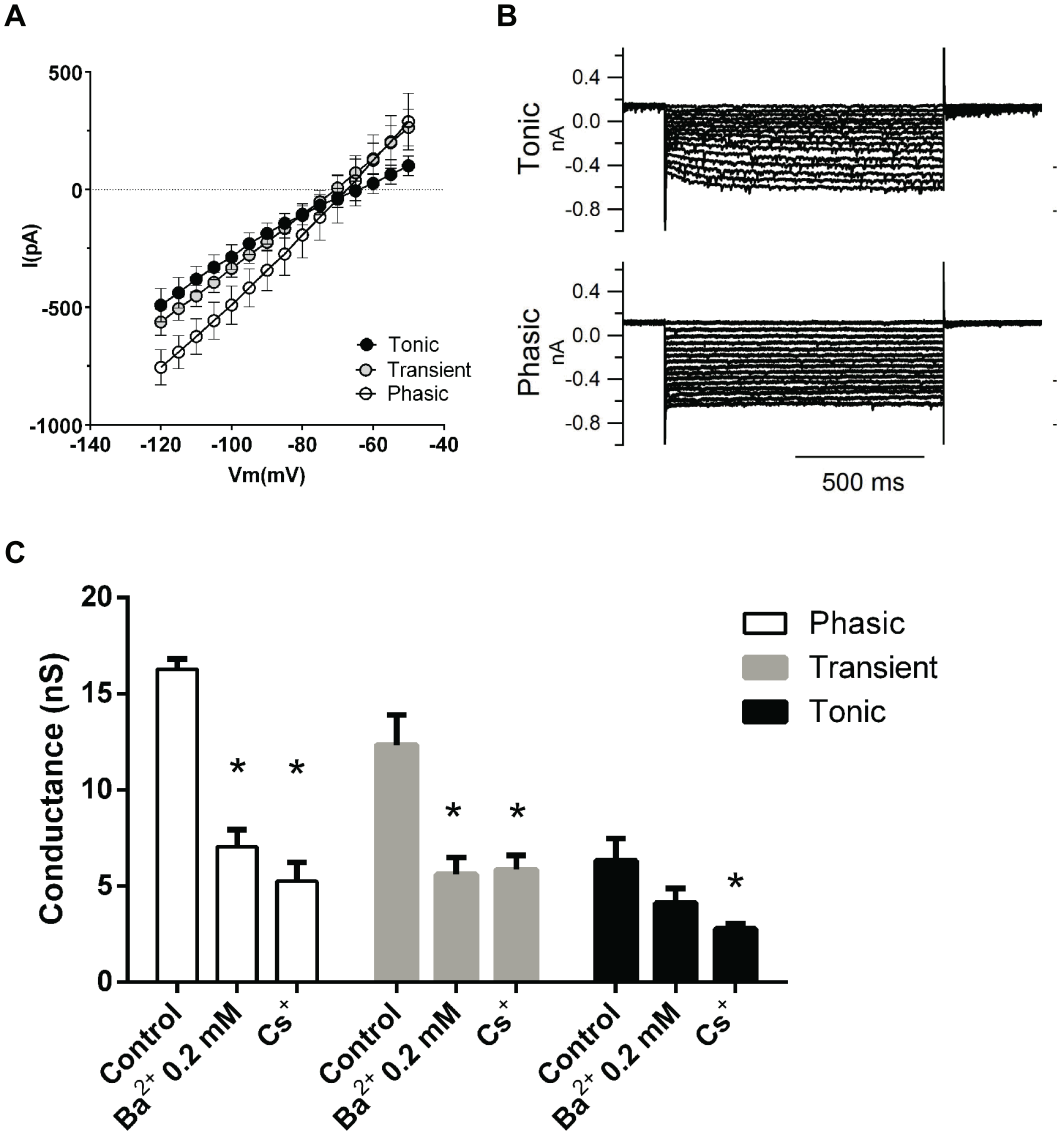
**Subthreshold conductances of the three neuronal types. (A)** IV relationships of the subthreshold currents from tonic (*n* = 3), transient (*n* = 6), and phasic neurons (*n* = 6). **(B)** Example of subthreshold currents from a tonic and a phasic neuron. **(C)** Effect of the sequential application of BaCl_2_ (0.2 mM) and CsCl (3 mM) on the subthreshold membrane conductance of phasic, transient, and tonic neurons (*n* = 5, 6, and 3, respectively). ^∗^*p* < 0.05 compared with the control condition.

Since phasic neurons were found to have a lower membrane resistance than either tonic or transient neurons, we predicted that they might also express larger subthreshold and/or leak conductances, when measured in voltage-clamp (**Figure 4A**). Indeed, we found that, accordingly, subthreshold membrane currents were bigger in phasic neurons and presented a linear IV from –120 to –40 mV, except in tonic neurons were we observed a slight inward rectification (**Figure 4B**). Subthreshold membrane conductances were bigger in phasic neurons (11.0 ± 1.3 nS; *n* = 15) and in transient neurons (9.2 ± 1.6 nS; *n* = 10) than in tonic neurons (5.0 ± 0.7 nS; *n* = 7; *p* = 0.04, One-Way ANOVA; *p* < 0.05; Newman–Keuls multiple comparison test).

We then asked which would be the background conductances of these different neuronal types. Several channels can be responsible for the resting membrane conductance, like the HCN channels responsible for the inward cationic current (*I*_h_; Biel et al., 2009), the Kir channels responsible for the inwardly rectifying potassium current (Hibino et al., 2010) and a variety of voltage-independent leak channels mostly permeable to potassium (Enyedi and Czirják, 2010). Tonic neurons exhibited in average a small inward rectification at –70 mV (**Figure 4A**), suggesting the presence of inward rectifying potassium (*I*_Kir_) and/or cationic currents (*I*_h_). Additionally most tonic neurons exhibited a slow-activating inward *I*_h_-type current that could be elicited by hyperpolarization (**Figure 4B**), which was inhibited by applying a blocker of *I*_h_ currents (3 mM Cs^2+^; not shown). In addition most tonic neurons presented a depolarization sag of the membrane potential of 4.5 ± 1.8 mV (*n* = 6), when hyperpolarized, which is attributed to the activation of h current which was rarely seen in transient and phasic neurons. This sag is inhibited by the *I*_h_ inhibitors ZD7288 (20 μM) and CsCl (3 mM; not shown) confirming it is produced by *I*_h_. So it seems that inwardly rectifying currents, especially *I*_h_, are differentially expressed in tonic neurons.

To test the contributions of *I*_h_ and *I*_Kir_ to the resting conductance of these neurons we applied sequentially BaCl_2_ 0.2 mM to inhibit *I*_Kir_ currents and CsCl 3 mM to block *I*_h_. Application of 0.2 mM Ba^2+^ produced a significant decrease in membrane conductance only in phasic and transient neurons (phasic: 16.3 ± 0.5 nS, to 7.0 ± 0.9 nS, *n* = 5; transient: 12.3 ± 1.5 nS to 5.6 ± 0.8 nS, *n* = 6; *p* < 0.05 One-way ANOVA, Newman–Keuls multiple comparison test) but not in tonic neurons (6.3 ± 1.1 nS to 4.2 ± 0.7 nS, *n* = 3; **Figure 4C**). However, the current that was affected by BaCl_2_ 0.2 mM did not have the inward rectification typical of *I*_Kir,_ (not shown), and we conclude that Ba^2+^ was largely inhibiting a fraction of a leak potassium current (Day et al., 2005).

We subsequently applied CsCl 3 mM to test the presence of *I*_h_. CsCl 3 mM decreased membrane conductance in tonic neurons (to 2.8 ± 0.2 nS, *n* = 3 *p* < 0.05 One-way ANOVA, Newman–Keuls multiple comparison test) and did not affect significantly the membrane conductance of phasic and transient neurons (phasic: to 5.2 ± 1 nS, *n* = 5; transient: 5.9 ± 0.7 nS, *n* = 6; **Figure 4C**). We can conclude that *I*_h_ has a significant impact in the membrane conductance only in tonic neurons and that the current inhibited by 0.2 mM of BaCl_2_ has no characteristics of a potassium inwardly rectifying current.

**FIGURE 5:**
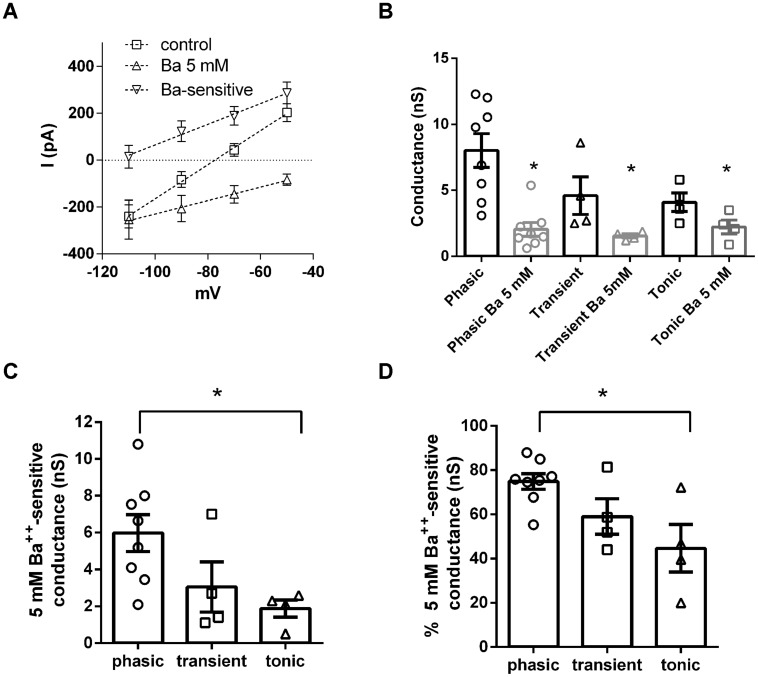
**Potassium leak conductances are differentially expressed in NCM neurons. (A)** IVs of the leak currents in control conditions, after Ba^2+^ 5 mM and the Ba^2+^ 5 mM-sensitive current in phasic neurons (*n* = 5). **(B)** Comparison of the effect of Ba^2+^ 5 mM in the subthreshold membrane conductance in phasic, transient, and tonic neurons (*n* = 8, 4, and 4, respectively, ^∗^*p* < 0.05 in comparison with the control condition). **(C)** Comparison of the Ba^2+^ 5 mM sensitive subthreshold membrane conductance in phasic, transient, and tonic neurons, ^∗^*p* < 0.05. **(D)** Comparison of the proportion of Ba^2+^ 5 mM subthreshold membrane conductance related to the total subthreshold conductance in phasic, transient and tonic neurons, ^∗^*p* < 0.05.

Another family of subthreshold currents are the potassium leak currents. Although potassium leak currents do not have a specific pharmacological inhibitor, they can be blocked by millimolar concentrations of BaCl_2_. We then compared the role of potassium leak conductances on the subthreshold membrane conductance of NCM neurons by inhibiting these currents with BaCl_2_ 5 mM (**Figure 5A**). Application of BaCl_2_ (5 mM) decreased significantly subthreshold conductances in all three neuronal types tested (phasic: from 8.2 ± 1.3 nS to 2.0 ± 0.5 nS, *n* = 8; transient: 4.6 ± 1.5 nS to 1.5 ± 0.15 nS, *n* = 4; tonic: 4.1 ± 0.7 nS to 2.2 ± 0.5 nS, *n* = 4, *p* < 0.05, transient and tonic; *p* < 0.01, phasic, Paired *t*-test; **Figure 5B**). The remaining conductances were not significantly different among all neuronal types (*p* = 0.7, One-Way ANOVA). Accordingly the 5 mM Ba^++^-sensitive current was bigger in phasic neurons (5.9 ± 1 nS) than in transient and tonic neurons (transient: 3.0 ± 1.3 nS; tonic: 1.9 ± 0.5 nS; *p* < 0.05, One-Way ANOVA, Newman–Keuls multiple comparison test; **Figure 5C**). Moreover the proportion of the Ba^++^-sensitive current was bigger in phasic than transient than tonic neurons (phasic: 74.8 ± 3.6%; transient: 59.0 ± 8%; tonic: 44.6 ± 11%; *p* < 0.05, One-Way ANOVA, Newman–Keuls multiple comparison test; **Figure 5D**).

Consistent with being a potassium conductance, the 5 mM Ba^++^-sensitive background conductance had a reversal potential of –98.6 ± 5 mV (*n* = 8) a value close to our theoretical potassium Nernst equilibrium potential (*E*_K_ = –105 mV). Showing that this conductance is important for maintaining RMP, after treatment with Ba^2+^ the membrane conductance had a higher reversal potential (–35.6 ± 5 mV in comparison to –82.5 ± 2 mV in control conditions; *p* < 0.0001; *n* = 8; **Figure 5A**).

We conclude that while the background currents of tonic neurons can have a component from *I*_h_, the ones from phasic and transient neurons seen to be composed mostly of linear leak currents, and that the magnitude of this Ba^++^-sensitive potassium leak current produces the differences in membrane input resistance observed among the three different neuronal types in the NCM.

### Voltage-activated Potassium Currents are Similar in the Three Neuronal Types

**FIGURE 6:**
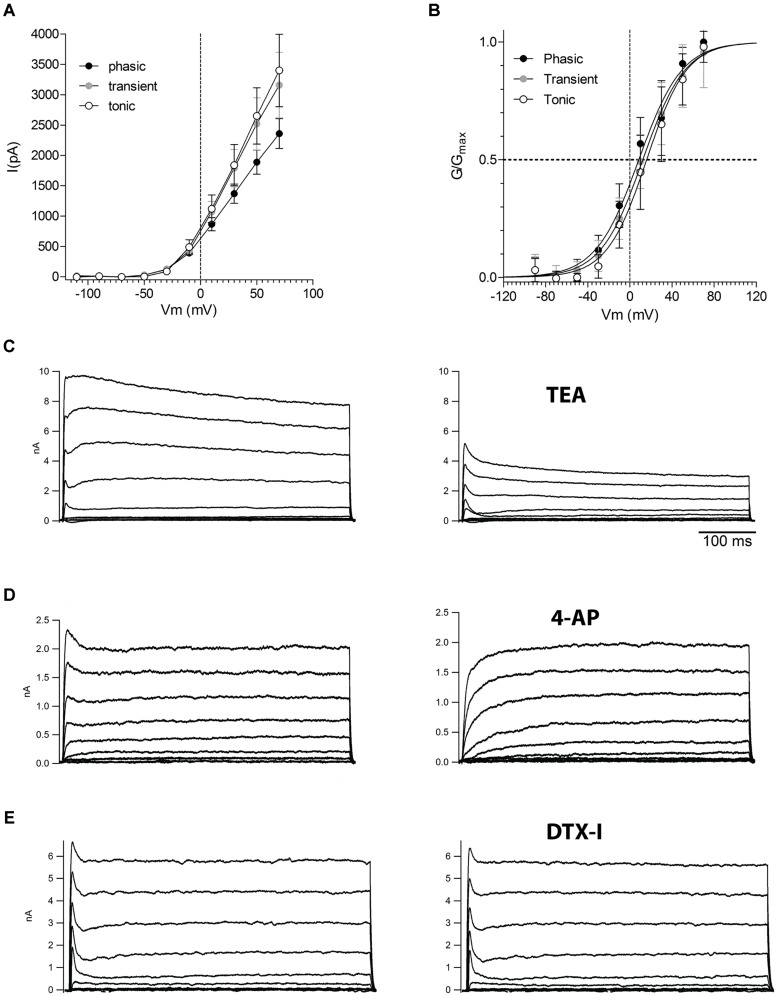
**Voltage-activated potassium currents in the three neuronal types in the NCM. (A)** Current-voltage (IV) relationships of the steady-state voltage-activated potassium currents in phasic, transient, and tonic neurons (*n* = 13, 11, and 11, respectively). **(B)** Activation curves of the voltage-activated potassium currents in phasic, transient, and tonic neurons. The data were fitted with a Boltzmann function. **(C)** Representative example of voltage-activated potassium currents before and after the application of TEA 5 mM (example from a phasic neuron). **(D)** Representative example of voltage-activated potassium currents before and after the application of four AP 5 mM (example from a tonic neuron). **(E)** Representative example of voltage-activated potassium currents before and after the application of DTX-I 100 nM (example from a phasic neuron). Recordings made in the presence of TTX. Voltage steps are from –70 to 70 mV in 20 mV steps.

Because phasic firing was the most common and stereotyped pattern of firing found in NCM neurons we then decided to investigate the ionic mechanisms responsible for generating the phasic firing pattern in NCM neurons. Phasic firing is usually attributed to low threshold activated potassium currents, so we hypothesized that phasic neurons would have VAKCs with lower activation potentials than transient and tonic neurons. Also differences in magnitude, activation and inactivation features of voltage-activated potassium currents might be responsible for the differences in firing observed in NCM neurons. We then recorded VAKCs under voltage-clamp, and in the presence of tetrodotoxin (TTX; 1 μM) to block voltage-activated sodium currents. We found that all three neuronal types exhibit large voltage-activated outward currents (**Figure 6A**) with IV relationships that have overall similar shapes and magnitudes (measured at the peak; (*I*_K_@70 mV: Tonic: 3.4 ± 0.6 nA; Transient: 3.2 ± 0.5 nA; Phasic: 2.4 ± 0.2 nA, *p* = 0.1; one-way ANOVA; *n* = 10, 11, and 11, respectively; **Figure 6A**). We also observed no significant differences in the time-course of VAKC activation among the three neuronal types (time constant of a single exponential function fitted to the rise time of the current at 30 mV: tonic: 2.3 ± 0.9 ms; transient: 2.3 ± 0.4 ms; phasic: 3.2 ± 0.8 ms; *p* > 0.05; One-way ANOVA).Contrary to our expectations, activation curves were typical of high-threshold VAKCs (activating around –20 mV; **Figure 6B**; *V*_1/2_: Phasic: 8.4 ± 1.6 mV; Transient: 12.3 ± 1.6 mV; Tonic: 16.0 ± 1.5 mV; *p* = 0.3; one-way ANOVA).

Application of TEA (5 mM) blocked most of the VAKCs in all cell types, leaving behind a small residual non-inactivating component and a fast-inactivating component (**Figure 6C**). The latter could be blocked by application of 4-aminopyridine (5 mM; **Figure 6D**). In many mammalian neurons, phasic firing is associated with the presence of dendrotoxin-sensitive low-threshold VAKCs (Brew and Forsythe, 1995; Dodson et al., 2002). However, application of dendrotoxin 1 (DTX-1; 100 nM), a specific inhibitor of low-VAKCs failed to inhibit any components of the VAKCs in phasic neurons (**Figure 6E**) corroborating the finding that NCM neurons are largely devoid of low-VAKC.

### Inhibition of Voltage-activated Potassium Currents does not Change the Phasic Firing Pattern

**FIGURE 7:**
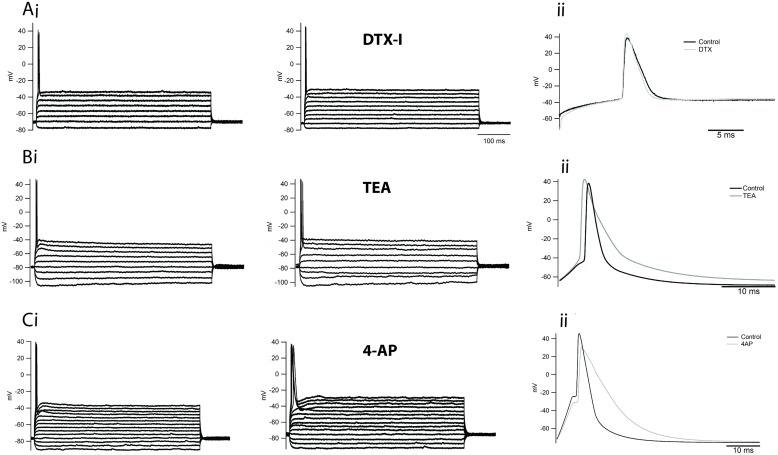
**Inhibition of delayed rectifiers, A-type and DTX-sensitive low-threshold potassium currents does not affect the firing of phasic neurons. (Ai)** Example of the firing of a phasic neuron before and after the application of DTX-I 100 nM. Expanded trace in **(Aii)**. **(Bi)** Example of the firing of a phasic neuron before and after the application of TEA 5 mM. Expanded trace in **(Bii)**. **(Ci)** Example of the firing of a phasic neuron before and after the application of 4-AP 5 mM. Expanded trace in **(Cii)**.

Phasic firing is usually associated with the expression of DTX-sensitive low-threshold Kv1.1 channels (Dodson et al., 2002) but our electrophysiology results suggest that phasic neurons likely do not express DTX-sensitive low-threshold VAKCs. Consistent with this observation, application of DTX-I to phasic neurons under current clamp, and in the absence of TTX, failed to alter either the phasic firing or the AP waveform of these cells (**Figure 7A**). To test the efficacy of our batch of toxin we tested the ability of our DTX-I to change the phasic firing of principal neurons of the medial nucleus of the trapezoid body (MNTB) from rats, to tonic firing. In accordance to already reported (Brew and Forsythe, 1995) DTX-I switched the firing of these neurons from phasic to tonic (not shown).

We therefore wondered whether other VAKC components might be responsible for limiting the firing of phasic neurons. To address this question, we first applied the broad VAKC antagonist TEA (5 mM) in the absence of TTX, which we had previously shown to block most of the delayed rectifying VAKC in phasic neurons, and compared the pattern of firing before and after drug application. Surprisingly, application of TEA 5 mM failed to alter the firing of phasic neurons (**Figure 7Bi**), only increasing the width of elicited APs (**Figure 7Bii**). Since Ca^2+^-activated potassium channels are also blocked by 5 mM millimolar TEA (Wulff et al., 2007), we next independently assessed the contribution of these channels to phasic firing by blocking the entry of calcium with a broad-range calcium channel blocker, cadmium chloride (CdCl_2_; 0.5 mM). Similar to the effects observed for TEA, CdCl_2_ application did not appreciably alter the firing of phasic neurons (not shown; *n* = 3), suggesting that Ca^2+^-activated potassium currents likely do not participate in the regulation of phasic firing.

A-type potassium currents are capable of modulating neuronal firing rates in several neuronal cell types (Connor and Stevens, 1971; Kim et al., 2005), and thus could potentially limit the firing of phasic neurons. We then applied four-AP (5 mM), which we had previously shown blocks A-type currents in NCM neurons (**Figure 6D**) to assess the role of this current in regulating phasic firing. At this concentration, four-AP also failed to alter the firing pattern of these neurons (**Figure 7Ci**), but it broadened the AP waveform (**Figure 7Cii**). We note that combining TEA and four-AP also failed to alter the firing pattern of phasic neurons (*n* = 4; not shown).

### Leak Currents Regulate the Firing Pattern of NCM Phasic Neurons

Since phasic neurons were found to have a very large subthreshold or leak conductance compared to other NCM neurons (**Figure 3**) producing a lower *R*_i_, and VAKCs do not regulate their phasic firing behavior (**Figure 7**), we wondered whether the leak currents might be responsible for regulating the phasic firing of this cell type.

**FIGURE 8:**
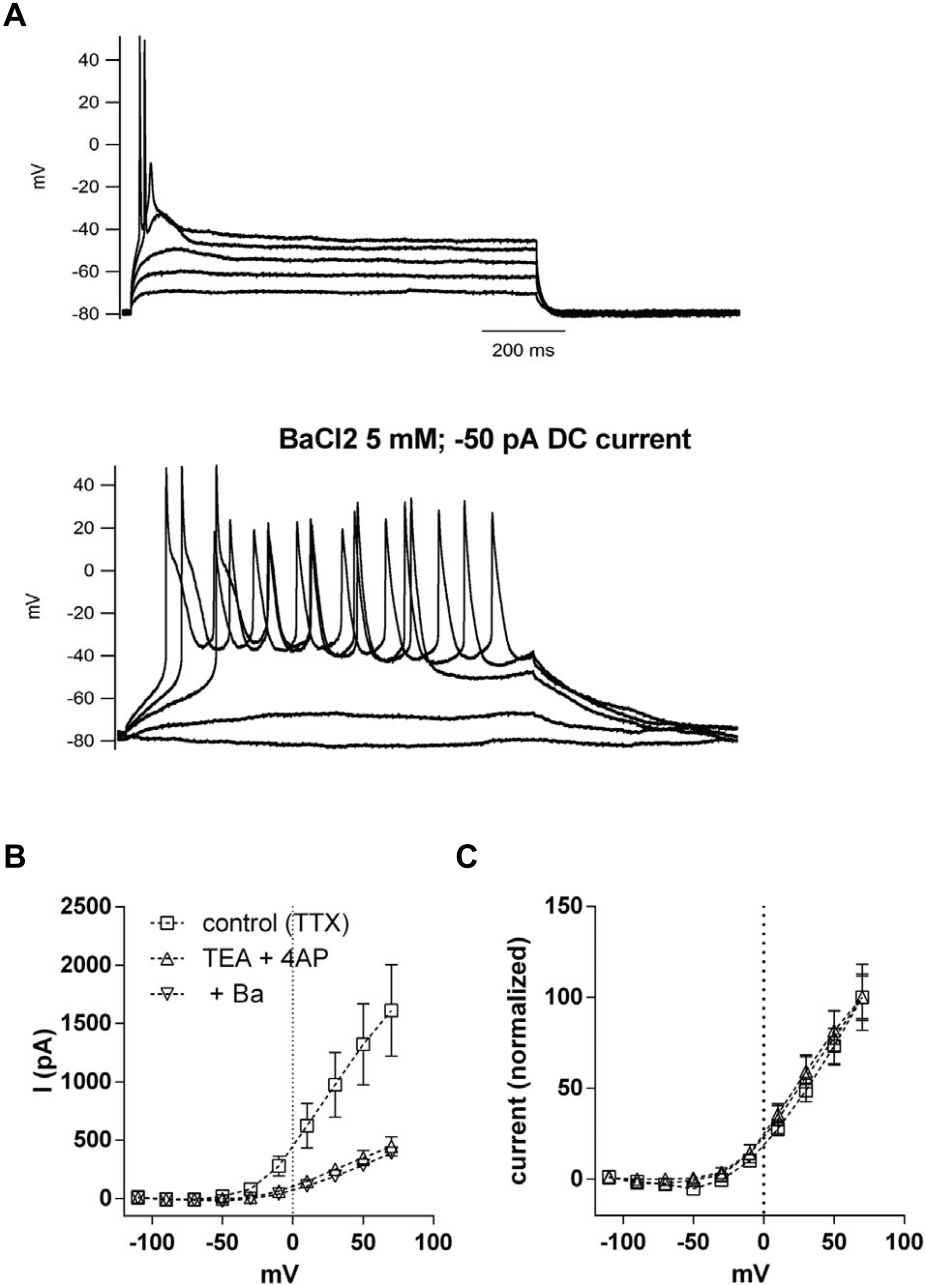
**Inhibition of potassium leak conductances by Ba^2+^ 5 mM changes phasic firing to tonic firing. (A)** Example of the firing of a phasic neuron before and after the application of BaCl_2_ 5 mM. Negative DC current was injected in the presence of Ba^2+^ to repolarize the RMP. **(B)** Current–voltage (IV) relationships of the voltage-activated potassium currents in phasic neurons before and after successive application of TEA (5 mM)/four-AP (5 mM) and BaCl_2_ (5 mM). *n* = 7. **(C)** Data from **(B)** normalized to the peak of current at 70 mV.

We then tested if BaCl_2_ 5 mM could transform a phasic neuron into a tonic one. In current clamp, the application of BaCl_2_ (5 mM) to phasic neurons resulted in a strong and significant membrane depolarization (–79.0 ± 2.1 mV to –39.1 ± 3.5 mV; *n* = 5; *p* < 0.001, paired *t*-test;), accompanied by a marked increase in membrane input resistance, as evidenced by an increase in the membrane time constant (10.8 ± 2.2 ms to 42.8 ± 5 ms; *n* = 5; *p* < 0.001; paired *t*-test). When we applied a negative DC current to bring the membrane potential back to original levels, depolarization steps produced tonic firing behavior that was not previously observed in the absence of Ba^2+^ (**Figure 8A**). These results suggest that leak currents in NCM neurons not only set both the RMP and membrane input resistance, but also influence patterns of neuronal firing in phasic neurons

But, because BaCl_2_ at the concentration of 5 mM is known to be a potent blocker of VAKCs (Gutman et al., 2005), and in NCM phasic neurons it also blocked most of the VAKCs (not shown) we decided to rule out the possibility that a blockade of a TEA/4AP-insensitive component of the VAKC by BaCl_2_ could affect firing. We then applied a cocktail of TEA/4AP to block VAKCs, and then added BaCl_2_ 5 mM to block any remaining VAKCs. Our results indicate the subsequent application of BaCl_2_ 5 mM did not further inhibit VAKCs (**Figure 8B**). The remaining current also has a similar IV relationship than the control current when normalized to the maximum current (**Figure 8C**), showing that differences in the IVs of the TEA/4AP/Ba-resistant VAKCs are not responsible for the transition from phasic to tonic firing. Thus, we conclude that the effects of BaCl_2_, at the concentration of 5 mM, on phasic neuron firing properties must be caused by the blockade of Ba^2+^-sensitive leak conductances.

### Introducing an Artificial Leak Conductance after BaCl_2_ Rescues the Phasic Firing Pattern

**FIGURE 9:**
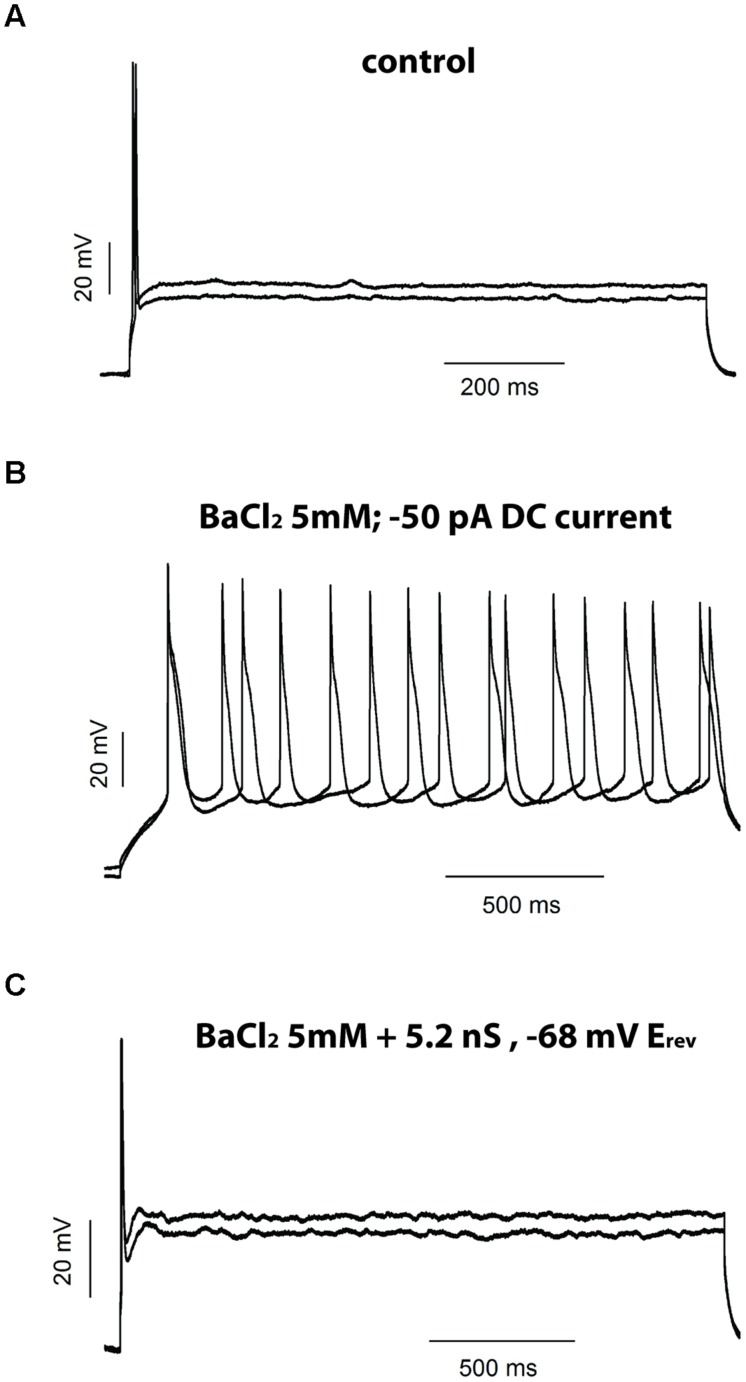
**Injection of an artificial leak current rescues the phase firing after BaCl_2_ 5 mM.** Example of the firing of a phasic neuron before **(A)** and after **(B)** application of BaCl_2_ 5 mM and after injection of an artificial leak current in the presence of BaCl_2_ **(C)**.

Our data thus far suggest that Ba^2+^-sensitive background leak currents, and not VAKCs, are likely responsible for restricting repetitive firing in phasic NCM neurons. To directly test this hypothesis and eliminate any other non-specific effect of Ba^2+^, we used a dynamic-clamp technique to apply an artificial conductance that mimics the background leak currents sensitive to Ba^2+^. If a linear leak conductance can restrict the firing of the phasic neurons then applying a “pure” leak conductance, after blockage of the endogenous one by Ba^2+^, will revert the effect of Ba^2+^ to induce tonic firing, ruling out any unspecific pharmacologically actions of Ba^2+^. For this we applied BaCl_2_ at 5 mM, measured the leak current it inhibited and applied a similar current using the dynamic-clamp. In the five phasic neurons tested with this protocol, Ba^2+^ 5 mM increased RMP and produced tonic firing (when the neuron was repolarized with DC current; **Figures 9A,B**). The injection of an artificial leak conductance with the same magnitude and reversion potential to the conductance inhibited by Ba^2+^ was able to not only hyperpolarize the membrane potential back to values similar to resting but also to switch back the cell’s firing state from tonic to phasic (**Figure 9C**). These results show that background leak currents, most likely those conducted by potassium leak channels, are capable of shifting neurons from a repetitive firing pattern to a phasic firing state.

### Computational Modeling Supports a Role for the Potassium Leak Conductance in Determining Phasic Firing

**FIGURE 10:**
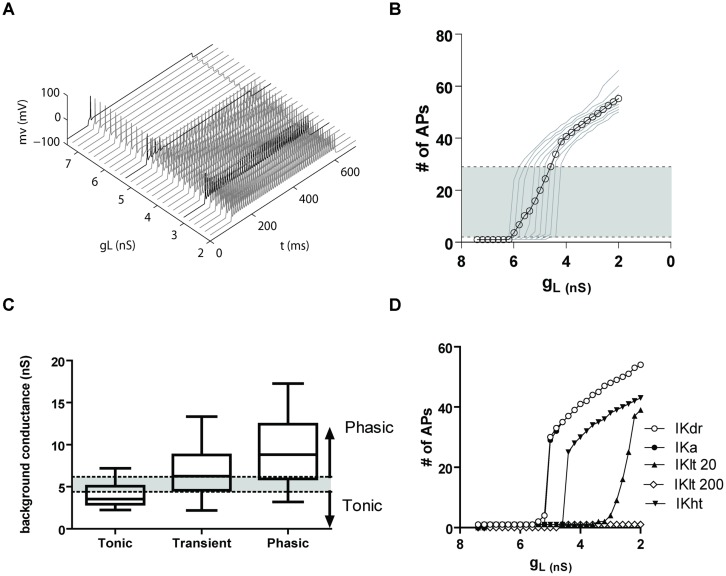
**(A)** A computer model shows that a potassium leak conductance is sufficient to switch firing from tonic to phasic. **(A)** Firing of a model phasic NCM neurons with a range of potassium leak conductances (*g*L), from 7.6 to 2 nS. The darker traces show examples of phasic, transient and tonic firing. **(B)** Graph showing the number of APs fired by the model vs. the leak conductance. The light traces represent different IKdr levels of conductance (from 60 to 140% of the average IKdr conductance of a phasic neuron), and the open symbols the average. The shaded area represents the transient firing expression, being phasic firing expressed below and tonic firing above it. **(C)** Comparison of the background conductances of the different neuronal types obtained from the data in **Figure 3B** with the data from the model. The box and whisker plot represents the 25–75% percentiles of the experimental data, and the bars the minimum and maximum points. The shaded area shows the levels of background conductance producing transient firing and the arrows the conductance levels producing phasic (above) and tonic (below) firing in the model. **(D)** Plot showing of the number of APs fired vs. leak conductance in a model with the average VAKC from a phasic neuron (IKdr) and adding an A-type potassium current (IKa), a 20 nS low-threshold potassium current (Klt 20), 200 nS of IKlt (IKlt200) and a high-threshold potassium current (IKht).

To better understand how potassium leak and voltage-activated currents regulate the firing properties of NCM phasic neurons, we generated a computer model of an NCM-like neuron using data from a phasic firing bushy neuron model found in the mammalian ventral cochlear nucleus (VCN; Rothman and Manis, 2003), but with a delayed rectifier (*IK*_DR_) potassium current with conductance and activation parameters obtained from NCM phasic neurons. Starting with a potassium leak conductance value (7.4 nS) similar to the mean leak conductance of phasic NCM neurons, we progressively reduced it and measured the number of APs elicited by our model neuron in response to a depolarization step of 120 pA. Our model cell fired phasically when leak conductances were in the range of 7.4–5.6 nS, but switched to transient firing when the conductance was between 5.4 and 5.2 nS (**Figure 10A**). In this model, we noted that the window for transient firing was relatively narrow due to the fact that we did not vary the amplitude of the VAKC conductance. Since VAKC conductance measurements vary considerably across the population of phasic neurons we repeated the simulation using different levels of VAKCs (varying ±40% from the central mean). As predicted, varying the amplitude of the VAKC broadened the window for transient firing (**Figure 10B**). Importantly, the levels of background leak conductance required to switch firing mode corresponded well with the measured values for tonic and phasic neurons, and showed reasonable overlap with the transient neurons (**Figure 10C**). Thus, our model shows that even in the absence of low-threshold K^+^-currents, phasic neurons can be switched from tonic to phasic firing by simply changing the amplitude of the potassium leak conductance.

To determine the extent to which leak currents control firing in the presence of other K^+^-currents, and assess whether other K^+^-currents might also increase the likelihood of transient firing, we next compared the effects of adding additional potassium conductances to our model. When we added a transient *IK*_A_ (125 nS, obtained from a model of a fusiform cell from the dorsal cochlear nucleus, Kanold and Manis, 2001), we were surprised to find that even at very large conductance values, *IK*_A_ current had almost no influence on the effect of the leak conductance on firing (**Figure 10D**). We also added a low-threshold potassium current (*IK*_LT_) derived from the Rothman and Manis (2003) model of a bushy cell. Using their original conductance of 200 nS, we observed that the *IK*_LT_ effectively prevented both transient and tonic firing at all levels of leak conductance (**Figure 10D**). However, when the IK_LT_ conductance was reduced to 20 nS, we observed the reappearance of both transient and tonic firing, associated with a less abrupt transition between the two modes of firing (**Figure 10D**). We note that the window for transient firing was biased toward the lower leak conductances expressed by tonic neurons. Finally, incorporating a 150 nS high threshold potassium current from the bushy cell model of Rothman and Manis (2003) did not alter the sharp transition from phasic to tonic, but did right-shift the transition point (**Figure 10D**). Thus, our model demonstrates that, with the exception of high levels of a *IK*_LT_ conductance, other voltage-activated potassium conductances do not appreciably interfere with the role that leak conductances play in regulating phasic firing.

## Discussion

The NCM is an auditory processing forebrain area of songbirds that plays important roles in the recognition, discrimination, and memorization of birdsong. Here we have identified basic neuronal firing modes of NCM neurons and characterized their membrane properties and potassium currents. More importantly, our results demonstrate a novel role for leak potassium conductances in the regulation of neuronal firing.

### Neuronal Firing Diversity in NCM and Implications for the Auditory Processing of Birdsong

Our results provide evidence for at least three distinct neuronal firing patterns in NCM in response to sustained depolarization: phasic, transient and tonic. We note that we found no evidence for fast-spiking, delay-spiking irregular spiking, or burst-spiking neurons, that are typical of the mammalian cortex (Ascoli et al., 2008), suggesting possible differences in the organization of cortical-like sensory processing systems in birds and mammals. Despite the apparent lower diversity in birds, the presence of discrete cell types suggests the existence of electrophysiological specializations that are important for processing different aspects of the auditory information.

Transient and tonic neurons presented substantial diversity in their AP waveforms and after hyperpolarizations. Additionally some transient neurons were very similar to phasic neurons, with a low input resistance, but firing two APs during the depolarization stimulus. The heterogeneity of firing of tonic and transient neurons is strong evidence that they embrace different neuronal types. Additionally it is possible that the boundaries between these categories might be more subtle (Battaglia et al., 2013). An alternative hypothesis would be that the differences in firing would not necessarily mean the existence of 3 fixed neuronal types. For example, modulation of leak currents by GPCRs (Mathie, 2007) could change the firing of NCM neurons, and maybe switch firing mode producing a “switchable” neuron that could change its mode of firing accordingly to the circumstances. On the other hand we found evidences of *I*_h_ only in tonic neurons, suggesting that there are specific channels expressed by these neuronal types. Further experiments combining electrophysiology, pharmacology, morphology and single-cell channel expression will be necessary for a complete characterization of the neuronal types in NCM. Nevertheless, our initial biophysical characterization represents the first attempt to characterize the NCM neurons and provides valuable information regarding the firing of these neurons.

Of the three neuronal firing types observed in NCM, phasic neurons were found to have the lowest input resistances (*R*_i_) and shortest spike-time latencies. Since a low *R*_i_ tends to minimize spike latency, phasic neurons might therefore be well-suited for precisely encoding the onset of a stimulus (e.g., song; Reyes et al., 1994; Golding et al., 1999; Oertel, 1999; Yang and Feng, 2007). Temporal firing properties are normally affected by *R*_i_, as attested for example by neurons in the auditory brainstem that are involved in timing processing and that have very low membrane resistance. This results in a short membrane time constant, allowing for a short spike-time latency and low variability in firing (Oertel, 1999). Preliminary data using several short depolarizations of different lengths delivered at high frequencies indicates that phasic neurons have less jitter of AP firing (data not shown), confirming their adaptation to precise firing.

In contrast, the tonic and transient neurons resemble more closely the tonic-chopper and adapting neuronal types that are typically found in the auditory brainstem (Feng and Lin, 1996; Rodrigues and Oertel, 2006; Yang and Feng, 2007), and fast adapting neurons in the mammalian cerebral cortex (Ascoli et al., 2008). It remains to be established if these differences constitute specific adaptations for responding to the temporal features of the zebra finch song.

NCM slices typically show strong spontaneous AP activity that drives GABAergic neurotransmission, and bursts of glutamatergic transmission (Pinaud et al., 2008; Dagostin et al., 2012). Surprisingly, the vast majority of the neurons recorded in the present study did not have spontaneous firing. This suggests that the spontaneous neurotransmission seen in NCM slices is either generated by a sparse, extensively connected neuronal population, or driven by input from adjacent areas present in the slices.

### Voltage-activated Potassium Currents do not Contribute to Phasic Firing in NCM

The zebra finch brain expresses a broad diversity of K^+^-channels genes, including at least one representative from each of the major subfamilies (Lovell et al., 2013) revealing that K^+^-channel genes families identified in mammals are broadly expressed in the avian brain. We first hypothesized that phasic firing would be created by selective expression of low-VAKCs in phasic neurons. However, we found that tonic, transient, and phasic neurons express similar VAKCs. Contrary to our expectations the TEA-sensitive current expressed in phasic neurons is activated in more positive voltages (*V*_50_ > 0 mV) and are non-inactivating delayed rectifying potassium currents similar in amplitude and activation and inactivation to the VAKCs of tonic and transient neurons. We also observed that four-AP sensitive fast-inactivating A-type potassium currents were expressed also in all three types. Because the currents associated with these channels are typically activated near the threshold for AP generation, they are particularly well-suited for regulating AP firing rates and fidelity (Jerng et al., 2004). We did not find evidence of a four-AP sensitive, slow inactivating VAKC (*I*_KD_; Mitterdorfer and Bean, 2002) because four-AP only inhibited the fast-activating component of VAKCs in NCM neurons.

To our surprise, the broad target antagonists TEA and four-AP had no impact on phasic firing. They affected only AP waveform, as was expected (Mitterdorfer and Bean, 2002). This was an unexpected result since these antagonists act on a broad range of potassium channels controlling neuronal firing. For instance potassium channels composed from Kv1.1 subunits have been shown to regulate phasic firing and excitability in the mammalian (Dodson et al., 2002; Svirskis et al., 2002; Gittelman and Tempel, 2006) and avian (Fukui and Ohmori, 2003) auditory system neurons. These channels are inhibited by TEA and also by dendrotoxin-I, which also did not affect phasic firing of NCM neurons. Additionally we did not find any evidence of calcium-activated potassium currents controlling the firing of the phasic NCM neuron, since blocking calcium entry with cadmium did not affect phasic firing. We used 5 mM of the slow calcium buffer EGTA in the intracellular solution, but only fast calcium buffers as BAPTA are able to interfere with calcium activated potassium currents (Storm, 1987; Mitterdorfer and Bean, 2002), so fast calcium-dependent events are preserved in our recordings. On the other hand, it is very likely that calcium-activated potassium currents control after hyperpolarization and consequently modulate firing frequency of tonic and transient neurons.

### Potassium Leak Currents Regulates Phasic Firing

We found that most of the subthreshold conductances of NCM neurons consisted of potassium leak currents. Only tonic neurons presented evidences of other subthreshold conductance, the *I*_h_. Phasic neurons not only expressed a bigger potassium leak conductance, but also they expressed a bigger proportion of this conductance related to the total subthreshold conductance compared to tonic neurons. Transient neurons expressed intermediate levels of potassium leak conductances, which were not significantly different from both tonic and phasic neurons, in accordance with an intermediate mode of firing between phasic and tonic. Interestingly after blocking potassium leak conductances with Ba^2+^ 5 mM, the differences in membrane resistance of the three neuronal types disappeared, showing that the differential expression of these currents creates the gradient of membrane input resistances seen in the three types of NCM neurons.

Intriguingly, our results indicate that potassium leak conductances play a key role in regulating the phasic firing in NCM neurons. First identified in the giant squid axon, potassium leak currents are ohmic conductances that regulate RMP (Hodgkin and Huxley, 1952; Doan and Kunze, 1999). Leak currents are encoded by a large multigene family of 2-pore domain channels (KCNK; Enyedi and Czirják, 2010) whose main function is to maintain a hyperpolarized RMP. In NCM, we observed that potassium leak conductances not only regulate membrane potential, but also limit firing. Specifically, neurons that normally fire phasically, fire tonically in the presence of Ba^2+^, a leak conductance blocker. Moreover, we were able to rescue the phasic firing by directly injecting an artificial leak conductance equal to the current inhibited by Ba^2+^ showing that was a specific leak conductance blocked by Ba^2+^ that produces phasic firing in these neurons. Because Ba^2+^ does not permeate well trough calcium channels in the presence of physiological concentrations of calcium (Hess and Tsien, 1984), and the effects of Ba^2+^ on firing are reverted by simply injecting an artificial potassium leak conductance, we do not believe that Ba^2+^ permeating trough calcium channels in our experimental conditions could interfere with the firing of phasic neurons. Thus, our findings point to a novel function for potassium leak currents in regulating neuronal firing and generating neuronal diversity.

It has been previously shown that modulation of leak currents increase and decrease neuronal firing, respectively, in accordance with increases and decreases in RMP (Ries and Puil, 1999; Washburn et al., 2002; Berntson and Walmsley, 2008). We found, however, that phasic or tonic firing in NCM neurons was not correlated with RMP (**Figure 3**) and changing membrane potential in the same neuron did not alter phasic firing (not shown) discarding possible effects of membrane potential in AP firing. Additionally, after blocking leak currents with Ba^2+^, tonic firing was observed after hyperpolarizing the neuron to their previous RMP values, showing that tonic firing was not produced by a Ba^2+^-induced depolarization.

Consistent with these findings, our computer simulation demonstrates that by simply decreasing the amplitude of the leak conductance, neurons can switch states from phasic to tonic firing, largely independent of other VAKCs. Moreover, our simulation also revealed that the transition from phasic to tonic firing is very abrupt in response to lowering the leak conductance, suggesting that the window for switching between phasic and tonic firing (where transient firing occurs) is very narrow. We note, however, that combined variations in the levels of leak and VAKCs conductances can also increase the size of the window for transient firing, showing the importance of taking into account individual variations of specific conductances, rather than using only average values. It is not clear to us the advantages of using a background potassium conductance instead of a low-threshold potassium conductance in order to restrict firing, specially that birds do express this type of VAKCs in their brain (Lovell et al., 2013), but because KCNK channels can be strongly modulated by G-protein coupled receptors (Mathie, 2007) they conductance could be easily changed by neuromodulators, which could produce fast changes in the firing of these neurons.

In summary, our findings indicate that NCM is comprised of at least three basic neuronal types that can be readily discriminated based on differences in their patterns of evoked firing. Moreover, despite molecular and electrophysiological evidence that broad diversity of K^+^-channels are expressed in NCM neurons, leak-type potassium currents appear to be the primary determinant of the phasic firing mode. Thus, our study suggests a new physiological role for leak channels, and hints more broadly that these channels may be more prominent players in the generation of neuronal firing diversity than previously assumed.

## Author Contributions

AD, PL, CM, and RL designed the experiments; AD and RL performed experiments, AD and RL analyzed data; AD and MH performed computer and dynamic clamp simulations; AD, PL, MH, CM, and RL interpreted and discussed the data; AD, PL, MH, CM, and RL wrote the manuscript.

## Conflict of Interest Statement

The authors declare that the research was conducted in the absence of any commercial or financial relationships that could be construed as a potential conflict of interest.

## Footnotes

1 http://www.rtai.org

2 http://www.comedi.org
